# Nasal floor approach via maxillary vestibular access for removal of a high-positioned impacted supernumerary tooth in the maxillary anterior region: a case report

**DOI:** 10.3389/fdmed.2026.1942302

**Published:** 2026-10-19

**Authors:** Yanfang Zhong, Mengjie Dong

**Affiliations:** 1 Department of Stomatology, Jiande Hospital of Integrated Traditional Chinese and Western Medicine, Hangzhou, Zhejiang, China; 2 Department of Stomatology, Hangzhou Stomatology Hospital, Hangzhou, Zhejiang, China

**Keywords:** cone-beam computed tomography, impacted supernumerary tooth, nasal floor, maxillary anterior region, nasal cavity

## Abstract

High-positioned impacted supernumerary teeth near the nasal floor in the maxillary anterior region pose significant surgical challenges, as conventional labial or palatal approaches often require extensive bone removal and risk injuring adjacent teeth. This case report describes a 14-year-old female with recurrent swelling and discomfort in the maxillary anterior region. Cone-beam computed tomography (CBCT) imaging revealed a high-positioned supernumerary tooth adherent to the nasal floor bone plate. Via a maxillary vestibular incision, a bone window was created adjacent to the nasal floor with meticulous protection of the nasal mucosa, consistent with the modified surgical nomenclature. The tooth was successfully removed using a segmental sectioning technique. At the 3-month follow-up, healing was uneventful with no reported nasal symptoms or clinical evidence of infection or oronasal communication. This single case provides short-term technical feasibility evidence only and does not establish safety, comparative invasiveness, or a low complication risk.

## 1 Introduction

Supernumerary teeth are developmental anomalies with a prevalence of 1.5–3.5% in the permanent dentition (1), most commonly occurring in the maxillary anterior region (2). Impacted supernumerary teeth in this area may cause delayed eruption, crowding, root resorption, and cyst formation (1). Surgical removal is generally recommended, but treatment is challenging when the tooth is positioned high and close to the nasal floor (3). Conventional labial or palatal approaches often require extensive bone removal, increasing the risk of adjacent root damage, pulpal injury, and postoperative bone defects (4, 5). With the application of CBCT, the three-dimensional relationship between impacted teeth and surrounding structures can be accurately evaluated. In selected cases, a nasal floor approach via vestibular access may provide an anatomically direct surgical pathway in selected configurations; any comparative advantage remains unvalidated. This report describes the successful management of a high-positioned impacted supernumerary tooth adjacent to the nasal floor using this approach.

## 2 Case presentation

### 2.1 Patient information

A 14-year-old female presented with recurrent swelling and occasional pain in the maxillary anterior region for more than one year. She had no history of systemic disease and no family history of supernumerary teeth. Her concern about appearance and chewing prompted orthodontic evaluation. Additional medical, family, and psychosocial details were not documented.

### 2.2 Clinical findings

Extraoral examination revealed no facial asymmetry. Intraoral examination showed crowding in the right maxillary anterior region, with labial displacement of tooth 13. Tooth 22 was lingually displaced. Slight labial alveolar bone expansion was observed, without redness or swelling of the vestibular mucosa. No significant percussion tenderness was noted (Figure 1a).

**Figure 1:**
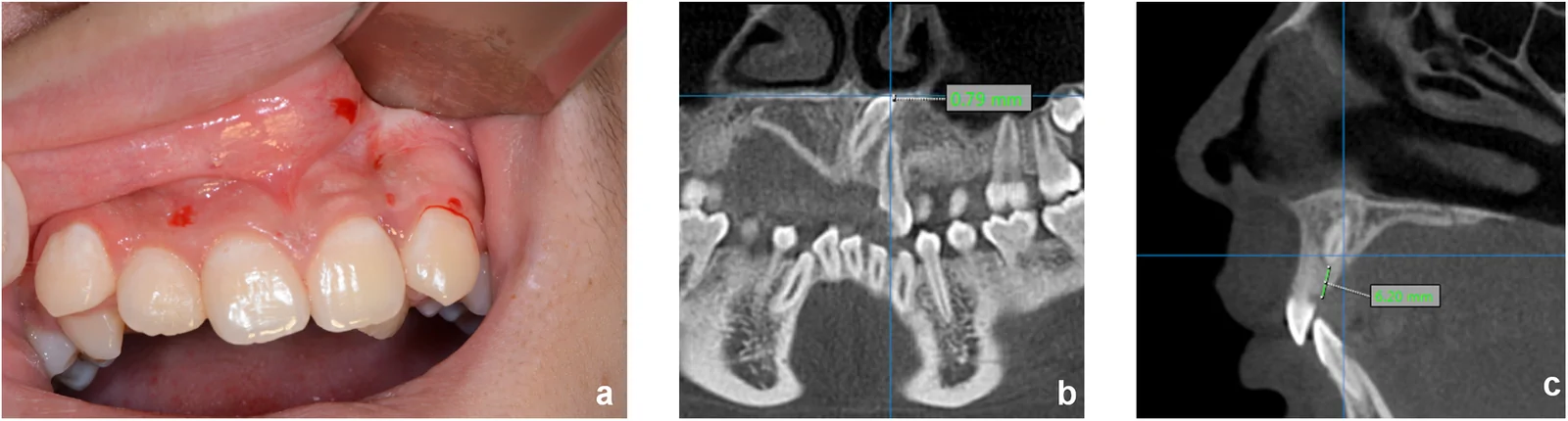
Preoperative examination. **(a)** Preoperative intraoral photograph. **(b,c)** Preoperative CBCT images.

### 2.3 Diagnostic assessment

CBCT images were acquired using a CS 9,600 unit (Carestream Dental, Atlanta, GA, USA) at 90 kVp and 5 mA, with an exposure time of 8.0 s, a 5 × 5 cm field of view, and an isotropic voxel size of 0.20 mm. Multiplanar reconstruction and linear measurements were performed by the operating surgeon using CS 3D Imaging software. CBCT imaging revealed a well-defined tooth-like radiopaque structure located apical to the maxillary central incisor and closely adherent to the nasal floor bone plate. The supernumerary tooth was inverted and conical in shape, positioned between the nasal floor and the root apex of the central incisor (tooth 21). The surrounding bone was thinned but continuous. Quantitative CBCT measurements showed the supernumerary tooth was 6.2 mm superior to the labial alveolar crest, with a 0.8 mm thick nasal floor cortical plate separating the tooth crown from the nasal cavity. The shortest distance between the supernumerary tooth and the apex of the adjacent left central incisor was 1.9 mm. No cystic lesions or other odontogenic pathologies were identified, and no root resorption of adjacent teeth was observed (Figures 1b,c). In this patient, recurrent swelling and occasional pain, together with the three-dimensional relationship to the nasal floor and adjacent roots, supported the decision for surgical removal after discussion with the patient and legal guardian. Based on these findings, a diagnosis of a high-positioned impacted supernumerary tooth in the maxillary nasal floor region was established. The tooth-like morphology on multiplanar CBCT favored an impacted supernumerary tooth over an odontoma or another calcified odontogenic lesion; the absence of a cystic lesion argued against an associated dentigerous cyst. The main diagnostic challenge was defining its proximity to the nasal floor and adjacent roots for access planning.

### 2.4 Timeline

The clinical course is summarized in Table 1. The patient reported misaligned maxillary anterior teeth affecting aesthetics and chewing function for 5 years prior to presentation. Over the past 1 year, she developed recurrent swelling and occasional pain in the same region, prompting her to seek orthodontic evaluation. Initial CBCT examination at the first visit identified the high-positioned impacted supernumerary tooth. After informed consent, surgical removal was performed 1 week later. Sutures were removed 7 days postoperatively. Postoperative clinical follow-up at 3 months demonstrated favorable healing with no clinically observed complication at that visit.

**Table 1:** Timeline of care.

Time	Clinical course
5 years before presentation	Anterior tooth misalignment affected appearance and chewing.
More than 1 year before presentation	Recurrent maxillary anterior swelling and occasional pain.
First visit	Clinical examination and CBCT identified the high-positioned impacted supernumerary tooth; surgical planning and consent.
1 week after first visit	Removal through a nasal floor approach via maxillary vestibular access.
Postoperative day 7	Sutures removed.
Postoperative month 3	Clinical and radiographic follow-up showed healing without a clinically observed complication at that visit.

### 2.5 Therapeutic intervention

Considering the high position of the supernumerary tooth and its proximity to the nasal floor, a nasal floor approach via vestibular access was selected on the basis of the CBCT-defined anatomy, with the intention of limiting labial bone removal and contact with adjacent roots. The surgical plan for “nasal floor approach via vestibular access extraction of the supernumerary tooth” and associated risks were explained in detail to the patient preoperatively. After the patient fully understood, informed consent was obtained. Following successful local infiltration anesthesia, a trapezoidal incision was designed in the maxillary vestibular fornix, extending from the distal aspects of teeth 11 and 22 to the midline (Figure 2a). After incising the mucoperiosteum, the buccal mucoperiosteal flap was reflected superiorly to fully expose the nasal floor and the labial bone surface of the anterior maxillary teeth (Figure 2b). After exposing the nasal floor, the nasal mucosa was carefully dissected within the surgical field, and sterile gauze was placed for protection and cushioning (Figures 2c,d) to prevent iatrogenic injury to the nasal mucosa during the procedure. Local infiltration anesthesia was administered using 4% articaine with epinephrine 1:100,000 (Septanest; Septodont, Saint-Maur-des-Fossés, France).

**Figure 2:**
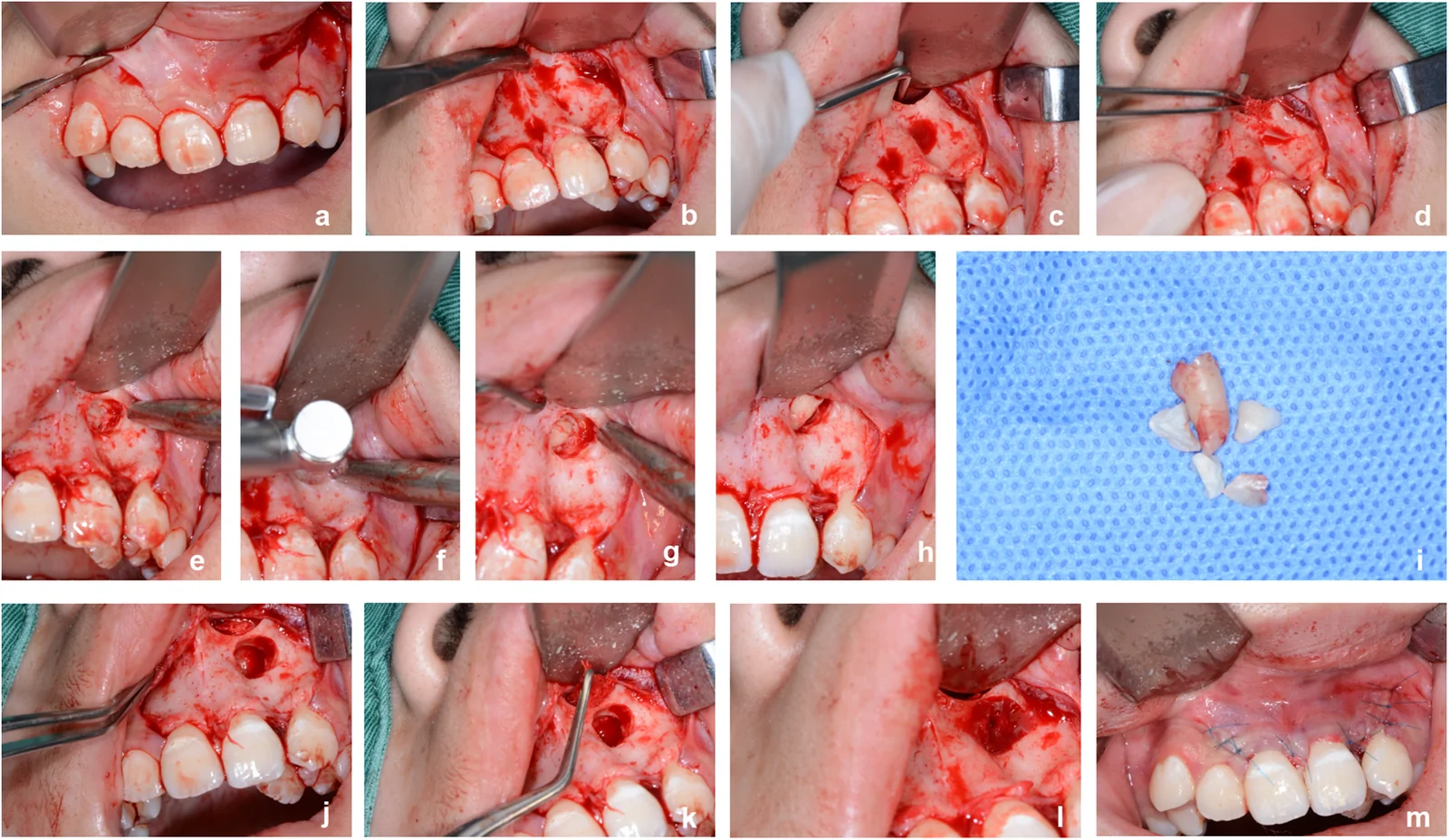
Surgical procedure. **(a)** Incision and flap elevation. **(b)** Determination of the surgical approach. **(c)** Dissection of the nasal mucosa. **(d)** Placement of sterile gauze for cushioning and protection. **(e–i)** Segmental extraction of the supernumerary tooth. **(j,k)** Removal of the sterile gauze. **(l)** Placement of medical collagen sponge into the extraction socket. **(m)** Wound suturing.

Under the guidance of CBCT localization, a bone window was created apical to teeth 21 and 22, near the nasal floor. The margins of the bone window were kept approximately 2 mm away from the apices of the adjacent teeth to reduce the risk of compromising the pulpal blood supply. Cortical osteotomy was performed using a Woodpecker Surgery-X with a US1 osteotomy tip in bone mode under continuous sterile-saline irrigation. Due to the tooth's tight integration with the surrounding bone, a tooth sectioning technique was employed: the tooth crown was sectioned longitudinally using a high-speed surgical handpiece and a long-shank tungsten-carbide fissure bur (ISO 010), after which the fragments were removed sequentially with a small straight elevator (Figures 2e–h), avoiding forceful traction that could damage the root surfaces of adjacent teeth or the nasal floor bone plate. The tooth and all fragments were completely removed (Figure 2i). The sterile gauze was retrieved (Figures 2j,k), revealing a thoroughly debrided surgical site with intact alveolar bone walls, no significant bone defect, and no communication with the nasal cavity. The area was irrigated thoroughly with normal saline. Intraoperatively, no nasal mucosa perforation was noted after gauze retrieval. Local hemostasis was achieved with gentle compression using sterile gauze for 5 min, with no active bleeding observed at closure. Postoperatively, the patient was prescribed amoxicillin 500 mg tid for 3 days for infection prophylaxis, ibuprofen 200 mg prn for pain, and instructed to avoid nose blowing, vigorous rinsing, and hard food in the surgical area for 2 weeks. Medical collagen sponge was placed into the bone window area (Figure 2l), the flap was repositioned and closed with 4-0 polypropylene monofilament sutures (PROLENE; Ethicon) using simple interrupted stitches (Figure 2m), concluding the procedure. The short amoxicillin course was prescribed as prophylaxis following bone-window osteotomy adjacent to the nasal floor, although no active infection or mucosal perforation was observed. Medication adherence and tolerability were not documented.

### 2.6 Follow-up and outcomes

At the 3-month follow-up, healing was uneventful. The 3-month intraoral photograph and panoramic radiograph showed no visible residual tooth-like radiopacity at the surgical site (Figures 3a,b). Pulp sensibility of teeth 11, 21, and 22 was assessed preoperatively and at 3 months by the same examiner using a refrigerant cold test (CanalPro Endo-Ice; COLTENE) and an electric pulp tester (Digitest 3; Parkell). Tooth 12 served as a control. At both assessments, teeth 11, 21, and 22 showed brief, non-lingering responses comparable with the control tooth and were negative to percussion. At 3 months, probing depths around teeth 11, 21, and 22 were 1–3 mm, with no bleeding on probing, suppuration, gingival recession, or pathological mobility. Percussion tests were negative. Static light-touch, directional-brush, sharp-blunt, and two-point discrimination tests were performed bilaterally over the upper lip and paranasal region. Responses were symmetrical, with no clinically detectable sensory deficit or patient-reported numbness at 3 months. No epistaxis, nasal obstruction, altered sensation, or persistent functional limitation was reported.

**Figure 3:**
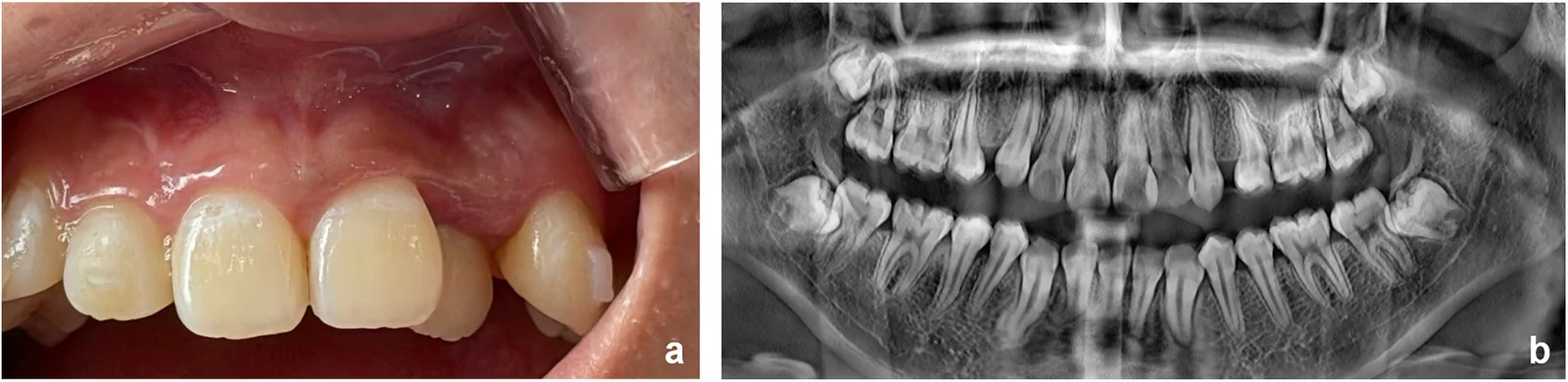
3-month follow-up examination. **(a)** Intraoral clinical photograph. **(b)** Panoramic radiograph.

### 2.7 Patient perspective

The patient reported that anterior tooth misalignment affected appearance and chewing and that recurrent swelling and occasional pain prompted evaluation. At 3 months, she reported no nasal symptoms, numbness, or persistent functional limitation. Her subjective account of the treatment experience and satisfaction was not documented.

## 3 Discussion

High-positioned impacted supernumerary teeth adjacent to the nasal floor are rare and present significant surgical challenges. Conventional labial or palatal approaches often require extensive bone removal, which may increase the risk of adjacent root injury and postoperative complications (6, 7). The nasal floor approach via vestibular access provided direct access in this patient while limiting the planned labial osteotomy; this observation cannot establish superiority over labial, palatal, combined, or transnasal approaches. Distinct from conventional labial approaches that often extend bone removal beyond the nasal floor margin, this technique is characterized by three refinements: (1) a bone window limited to the cortex adjacent to the nasal floor and planned approximately 2 mm from the adjacent tooth apices; (2) intentional dissection and gauze cushioning of the nasal mucosa prior to osteotomy; (3) an individualized, CBCT-informed technical suggestion to consider the spatial relationship among the tooth, nasal floor, labial cortex, and adjacent roots.

In this case, CBCT was essential for preoperative planning, as it clearly demonstrated the spatial relationship between the supernumerary tooth, the nasal floor, and adjacent tooth apices. This information was used to plan the bone-window location and the operative access. Nasal-mucosa protection and segmental tooth sectioning were technical steps intended to limit direct contact and force on adjacent structures; their effect on complication risk cannot be determined from this case. Although not suitable for all patients, the nasal floor approach via vestibular access may be considered as an unvalidated technical option in individual cases, particularly when the supernumerary tooth is tightly apposed to the nasal floor and labial access would require excessive bone removal. The limitations of this report include its short follow-up period and single-case design, highlighting the need for further studies with longer-term follow-up to evaluate sustained effects on adjacent tooth vitality, periodontal status, and bony remodeling. Literature specific to anterior maxillary supernumerary teeth supports three-dimensional localization and individualized access planning. A CBCT study of 487 patients found that 92% of supernumerary teeth were in the anterior maxilla and demonstrated the value of three-dimensional assessment (2). Observational pediatric series describe palatal, buccal, bicortical, and position-dependent approaches rather than a universal numerical rule (8, 9). For inverted teeth at or extending toward the nasal floor, a nine-patient prospective series and a separate case-based review described modified vestibular access with subperiosteal intranasal dissection (3, 6), while endoscopic transnasal removal has also been reported for teeth projecting into the nasal cavity (7).

Potential technical considerations include the tooth's three-dimensional relationship to the nasal floor, labial cortex, adjacent roots, and nasal mucosa; the presence of active infection or an oronasal communication; and whether another access route would require less disruption in the individual patient. Potential risks include transient oronasal communication, nasal mucosa injury, and adjacent root devitalization, all of which should be discussed with patients preoperatively.

## Key clinical message

For high-positioned impacted supernumerary teeth tightly adherent to the nasal floor, a vestibular-access nasal floor approach guided by CBCT was used in this patient with the aim of limiting labial bone removal. In this patient, nasal-mucosa protection and segmental tooth sectioning were technically feasible and were followed by uneventful healing over 3 months; safety, comparative invasiveness, complication risk, and durability require evaluation in larger series with longer follow-up.

## Data Availability

The raw data supporting the conclusions of this article will be made available by the authors, without undue reservation.
